# Edodin: A New Type of Toxin from Shiitake Mushroom (*Lentinula edodes*) That Inactivates Mammalian Ribosomes

**DOI:** 10.3390/toxins16040185

**Published:** 2024-04-10

**Authors:** Lucía Citores, Sara Ragucci, Claudia C. Gay, Rosita Russo, Angela Chambery, Antimo Di Maro, Rosario Iglesias, José M. Ferreras

**Affiliations:** 1 Department of Biochemistry and Molecular Biology and Physiology, Faculty of Sciences, University of Valladolid, E-47011 Valladolid, Spain; lucia.citores@uva.es; 2 Department of Environmental, Biological and Pharmaceutical Sciences and Technologies (DiSTABiF), University of Campania ‘Luigi Vanvitelli’, Via Vivaldi 43, 81100 Caserta, Italy; sara.ragucci@unicampania.it (S.R.); rosita.russo@unicampania.it (R.R.); angela.chambery@unicampania.it (A.C.); antimo.dimaro@unicampania.it (A.D.M.); 3 Laboratory of Protein Research, Institute of Basic and Applied Chemistry of Northeast Argentina (UNNE-CONICET), Faculty of Exact and Natural Sciences and Surveying, Av. Libertad 5470, Corrientes 3400, Argentina; claudiacgay@exa.unne.edu.ar

**Keywords:** protein synthesis (inhibition), pyridoxal phosphate (PLP), ribosome-inactivating protein (RIP), ribotoxin, rRNA N-glycosylase, shiitake (*Lentinula edodes*), toxin

## Abstract

Ribosome-inactivating proteins (RIPs) are a group of proteins with rRNA N-glycosylase activity that irreversibly inhibit protein synthesis and consequently cause cell death. Recently, an RIP called ledodin has been found in shiitake; it is cytotoxic, strongly inhibits protein synthesis, and shows rRNA N-glycosylase activity. In this work, we isolated and characterized a 50 kDa cytotoxic protein from shiitake that we named edodin. Edodin inhibits protein synthesis in a mammalian cell-free system, but not in insect-, yeast-, and bacteria-derived systems. It exhibits rRNA N-glycosylase and DNA-nicking activities, which relate it to plant RIPs. It was also shown to be toxic to HeLa and COLO 320 cells. Its structure is not related to other RIPs found in plants, bacteria, or fungi, but, instead, it presents the characteristic structure of the fold type I of pyridoxal phosphate-dependent enzymes. Homologous sequences have been found in other fungi of the class Agaricomycetes; thus, edodin could be a new type of toxin present in many fungi, some of them edible, which makes them of great interest in health, both for their involvement in food safety and for their potential biomedical and biotechnological applications.

## 1. Introduction

Ribosome-inactivating proteins (RIPs) are a group of proteins with rRNA N-glycosylase activity (EC 3.2.2.22) that catalyze the removal of a specific adenine (A4324 residue of rat) located in the sarcin–ricin loop (SRL) that is present in the large rRNA of eukaryotes and prokaryotes [1,2,3,4]. The SRL, together with the ribosomal stalk, forms the GTPase-associated center (GAC), which is the landing platform for the so-called translational GTPases (trGTPases), among which are elongation factors [5]. The adenine removed by RIPs is the most important base of the SRL since it contributes to establishing a cooperative interaction network, which stabilizes the active state of the trGTPases, promoting GTP hydrolysis required for the addition of amino acids into the nascent chain [5]. Therefore, the removal of such adenine results in the inhibition of protein synthesis and, consequently, in cell death.

RIPs are present in many angiosperm plants, some of them edible [1,2,3], and bacteria [4,6], while only one RIP has been obtained from algae [7] and six from fungi [8,9]. From a structural point of view, they have been classified into type 1 RIPs (a polypeptide chain with N-glycosylase activity) and type 2 RIPs (an A chain with the same enzymatic activity as type 1 RIPs and a B chain with lectin activity, linked by a disulfide bond) [2]. Type 2 RIPs, such as abrin or ricin, are highly toxic due to the presence of a B-chain that allows them to bind to cell membrane carbohydrates, thus accelerating the process of entry by endocytosis, whereas the toxicity of type 1 RIPs is much lower [2,10].

Other proteins that inhibit protein synthesis have been described in the fungal kingdom. Some have been designated as RIPs, but most are proteins lacking N-glycosylase activity [8]. Some of them are ribotoxins, which are specific rRNA endonucleases (EC 4.6.1.23) that catalyze the cleavage of the phosphodiester bond on the 3′ side of the G4325 residue of rat 28S rRNA [11,12]. Only five ribotoxins have been found in four ascomycetes species [11]. However, proteins with the same enzymatic activity have been recently isolated from the fruiting bodies of some edible basidiomycetes. Since these proteins have a different sequence and structure than the classical ribotoxins, they have been referred to as ribotoxin-like proteins (RL-Ps) [8]. A possible role as insecticides [11] and fungicides [13] has been proposed for ribotoxins and RL-Ps.

Although the biological function of RIPs is also unclear, RIPs have been implicated in plant defense against fungi, viruses, and insects [14,15].

Due to their translation-inhibiting and apoptosis-inducing activities, RIPs have been shown to be excellent tools for the development of selective antiviral and anticancer agents [15,16], and they have been used as a toxic part in several conjugates that have been tested in experimental therapies against various malignancies [16]. In agriculture, they have been used to obtain transgenic plants resistant to viruses, fungi, and insects [14,15].

Recently, an RIP termed ledodin was found in the shiitake mushroom [9]. This cytotoxic protein is a strong inhibitor of protein synthesis in mammals and shows N-glycosylase activity in the 28S rRNA SRL. However, it is inactive against ribosomes from insects, yeast, and bacteria. In addition, no relation has been found between the sequence and structure of ledodin and any protein with known function, although many fungi have homologous sequences in their genome [9].

By studying the cytotoxic and translation-inhibiting activities of shiitake, we found another cytotoxic protein that inhibits protein synthesis, which we named edodin. Apparently, edodin also displays N-glycosylase activity but has a completely different structure from that of ledodin or any RIP from plants or bacteria, presenting a structure that can be included in the fold type I of pyridoxal phosphate-dependent enzymes.

## 2. Results

### 2.1. Isolation of Edodin

**Figure 1:**
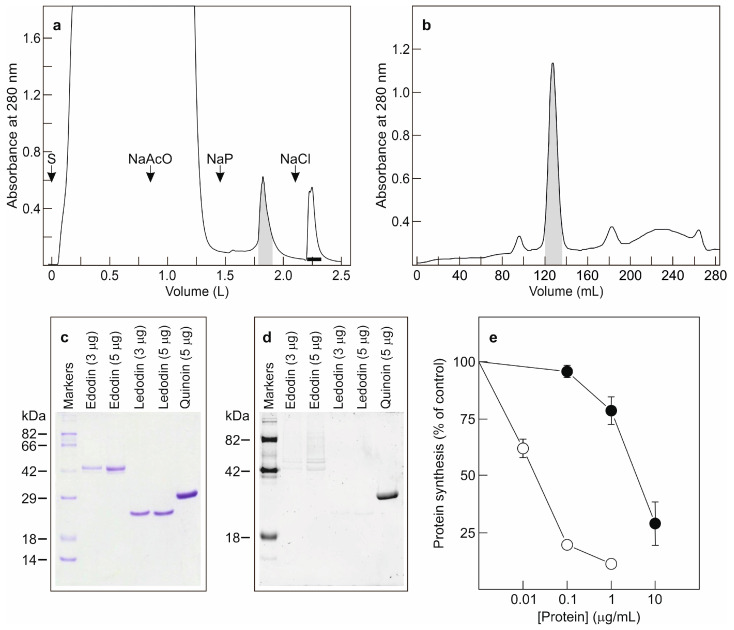
Purification of edodin from shiitake fruiting bodies. (**a**) The acidified extract was subjected to cation exchange chromatography on SP-Sepharose as described in Section 5. After the application of the sample (S) and washing with 10 mM sodium acetate (NaAcO), the column was eluted with 5 mM sodium phosphate (NaP) and then with the same buffer containing 0.5 M NaCl (NaCl). The shaded fraction was used to purify edodin, and the fraction marked with the horizontal bar contained ledodin. (**b**) The fraction eluted from the SP-Sepharose column with sodium phosphate was concentrated and chromatographed using a HiLoad^®^ 26/600 Superdex^®^ 75 pg column. The shaded fraction contained edodin. (**c**,**d**) Analysis of edodin by SDS-PAGE. Electrophoresis was performed as indicated in Section 5, and the gel was then stained with Coomassie brilliant blue (**c**) or by a glycoprotein staining kit. (**d**) Numbers indicate the corresponding size of standards in kDa. (**e**) Effect of edodin (closed circles) and ledodin (open circles) on protein synthesis. Translation assays were carried out using a cell-free system, as indicated in Section 5. The mean results ± SE of three experiments performed in duplicate are reported.

When using a method for the purification of ribosome-inactivating proteins (RIPs) from plants [17], an RIP named ledodin was isolated from shiitake fruiting bodies [9]. However, the purified protein did not account for all the cytotoxic and protein synthesis inhibitory activity of shiitake fruiting bodies, so we decided to investigate the presence of other proteins with these activities. For this purpose, an acidified extract was prepared as already described [9] and subjected to ion exchange chromatography on an SP-Sepharose column (Figure 1a). After loading the sample, the column was washed with sodium acetate and eluted, first with sodium phosphate and then with the same buffer but containing NaCl. The fraction eluted with NaCl contained ledodin (horizontal bar) [9]. The sodium phosphate-eluted fraction exhibited protein synthesis inhibitory activity; therefore, this protein peak was further purified by size exclusion chromatography. As shown in Figure 1b, Superdex 75 HiLoad chromatography revealed the presence of a main protein peak (shaded area) that contained a new purified protein named edodin. The estimated yield was 6.3 mg per 100 g of shiitake fruiting bodies. The protein obtained was analyzed by electrophoresis in polyacrylamide gels in the presence of SDS (SDS-PAGE) under reducing conditions. As shown in Figure 1c, edodin is a monomeric protein with a molecular weight of 50 kDa. Figure 1c,d compare proteins stained with Coomassie blue (Figure 1c) and those detected with a glycoprotein staining kit (Figure 1d). Quinoin, which is a glycosylated type 1 RIP [18], is shown as a control. It can be observed that edodin, like ledodin, did not show any staining, indicating that they are not glycosylated. We also tested edodin in a coupled transcription–translation in vitro assay using a rabbit reticulocyte lysate system, finding that edodin inhibits protein synthesis (Figure 1e), with an IC_50_ (concentration inhibiting 50% of protein synthesis) of 4 μg/mL (80 nM), 93 times higher than that shown by ledodin (Figure 1e), so it would be relatively high compared to other RIPs from plants and fungi [9].

### 2.2. Enzymatic Activity of Edodin

**Figure 2:**
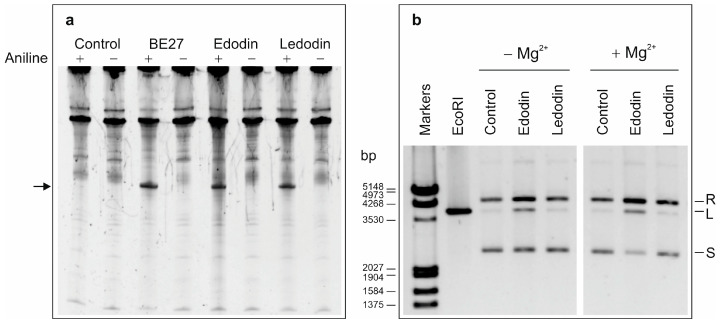
Enzymatic activities of edodin. (**a**) rRNA N-glycosylase activity of edodin in rabbit reticulocyte ribosomes compared to that of BE27 and ledodin; 5 µg of RNA isolated from untreated (control) or RIP-treated ribosomes were subjected to electrophoresis. The arrow points to the RNA fragment released as a result of RIP enzymatic activity followed by acid aniline cleavage (+). (**b**) Edodin and ledodin nicking activity on pCR2.1 DNA. Plasmid DNA samples (100 ng/10 μL) were incubated in the absence (control) or presence of 5 μg of toxin, without and with 5 mM MgCl_2_, as indicated in Section 5. One lane shows 200 ng of EcoR I-digested plasmid. R, L, and S indicate the relaxed, linear, and supercoiled forms of pCR2.1, respectively, and the numbers, the size of the markers (Lambda DNA-EcoR I/Hind III double digest), in base pairs.

Fungi produce proteins with different enzymatic activities that disable ribosomes to carry out protein synthesis and can be distinguished by ribosomal RNA electrophoresis [8]. Ribonucleases catalyze the hydrolysis of phosphodiester bonds causing the disappearance of ribosomal RNAs on electrophoresis [19]. Ribotoxins and RIPs are much more specific and affect the SRL, the difference being that ribotoxins are endoribonucleases and produce an RNA fragment (α-fragment) that can be detected by electrophoresis [8,20], whereas RIPs are N-glycosylases that do not produce a fragment unless the RNA is treated with aniline (β-fragment or Endo’s fragment) [8,21]. To determine which type of inhibitor edodin is, we performed Endo’s assay on rabbit reticulocyte ribosomes. As shown in Figure 2a, edodin causes, only after treatment with acid aniline, the release of a 460-nucleotide fragment identical to that produced by ledodin [9] and BE27 (an RIP obtained from beet leaves) [22] from rabbit reticulocyte ribosomes. This clearly indicates that edodin is an N-glycosylase that acts on the SRL of ribosomes. However, unlike BE27, and similar to ledodin [9], edodin was not active against ribosomes of mealworm, yeast, or the bacterium *Micrococcus lysodeikticus*, as evidenced by the absence of Endo’s fragment in the RNA electrophoresis (Figure S1a–c).

Many RIPs from plants, such as BE27, also possess adenine polynucleotide glycosylase (APG) activity, i.e., they can remove adenines from nucleic acids, either RNA or DNA [22,23], but, like ledodin [9], edodin did not show such activity (Figure S1d).

Finally, it has been shown that some RIPs cleave double-stranded supercoiled DNA, thus producing relaxed and linear molecules [22]. Therefore, we tested the nicking activity of edodin in the pCR2.1 plasmid in comparison to ledodin. In contrast to ledodin [9], edodin promoted the conversion of supercoiled pCR2.1 DNA into the relaxed and lineal forms (Figure 2b), activity that was enhanced by the presence of magnesium ions, as occurs with some RIPs from plants [22].

### 2.3. Cytotoxicity of Edodin and Antifungal Activity Assay

**Figure 3:**
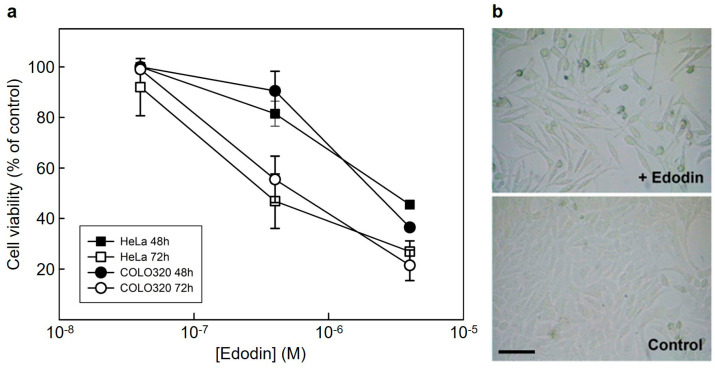
Cytotoxicity of edodin on cultured cells. (**a**) Effect of edodin on the viability of HeLa (squares) and COLO 320 (circles) cells. Cells were seeded in 96-well plates in a total volume of 100 μL of a complete medium containing various concentrations of edodin. Viability was evaluated after a 48 h (closed symbols) and 72 h (open symbols) exposure to the indicated concentrations of edodin using a colorimetric assay based on the cleavage of WST-1 tetrazolium salt, as indicated in Section 5. Data represent the mean ± SD of three experiments performed in duplicate. (**b**) Induction of apoptosis in HeLa cells by edodin. Phase contrast microscopy images 48 h after treatment of HeLa cells with 4 μM edodin. Bar, 50 μm.

Although plant type 1 RIPs are much less toxic than type 2 RIPs, they all exhibit cytotoxicity, with IC_50_ (concentration that reduces viability by 50%) for HeLa cells ranging from 2 to 34 µM [24]. The activity of edodin in cells was studied by incubating HeLa and COLO 320 cells with different concentrations of edodin for 48 and 72 h. As shown in Figure 3a, edodin reduced the viability of the two cell types in a concentration- and incubation time-dependent manner. In contrast to that reported for ledodin [9], edodin showed similar toxicity for the two cell types. Thus, at 72 h, the IC_50_ for HeLa cells was 0.35 µM (4-fold higher than that reported for ledodin), and the IC_50_ for COLO 320 cells was 0.59 µM (48-fold lower than that reported for ledodin). Moreover, when HeLa cells were treated with ledodin, morphological characteristics of apoptosis such as formation of apoptotic bodies, cell membrane blebbing, or cell shrinkage were observed [9], whereas few cells showed such features when treated with edodin (Figure 3b). Instead, the cells became elongated and lost contact, suggesting that pathways other than apoptosis were involved in edodin-mediated cell death.

To complete the spectrum of edodin toxicity, we also studied its effect on the growth of the fungus *Penicillium digitatum*. As shown in Figure S2, an edodin concentration of 100 µg/mL had no effect on fungal growth, whereas 15 µg/mL of BE27 or 1 µg/mL of α-sarcin reduced the growth of *P. digitatum* by 74 and 93%, respectively. This is in agreement with the result obtained with yeast ribosomes (Figure S1b), which can serve as a model of fungal ribosome.

### 2.4. Edodin Sequence

**Figure 4:**
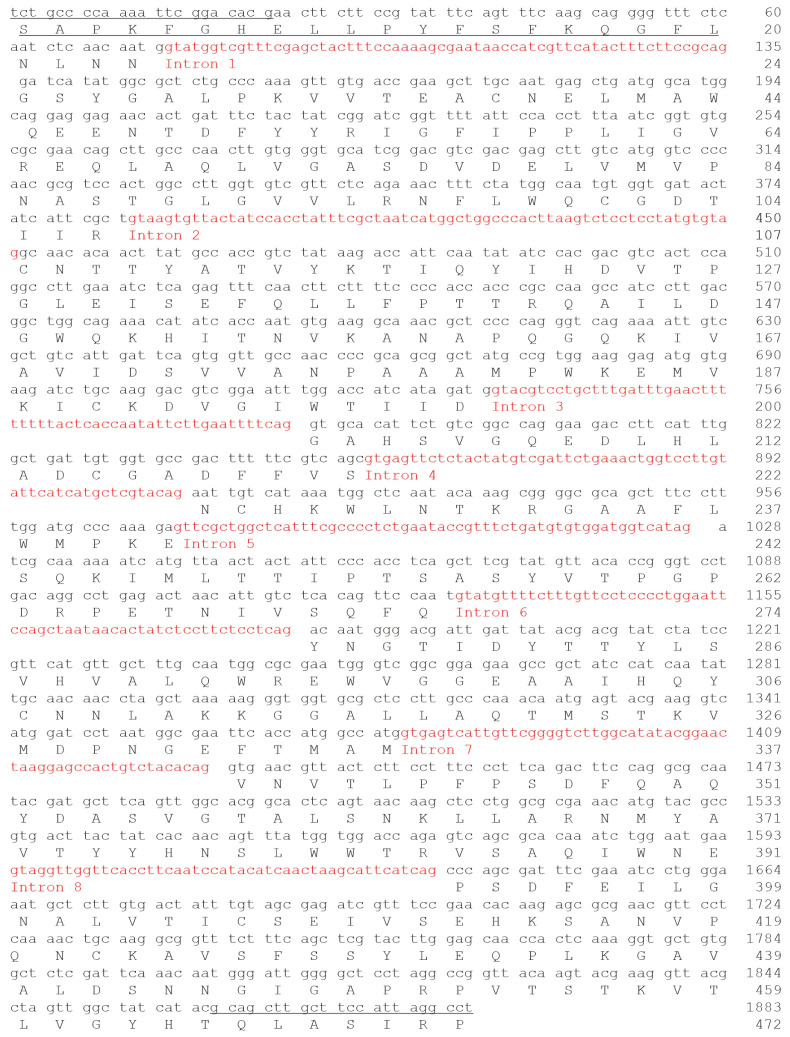
Sequence of the gene and amino acids of edodin. Primers and amino-terminal sequence are underlined, and introns are shown in red. Nucleotide and amino acid numbering is shown on the right. The DNA sequence for edodin was submitted to GenBank (accession number: PP003298).

To obtain information on the identity and structure of edodin, its N-terminal sequence was obtained by Edman degradation (Figure 4). Subsequently, the result was used to search for similar sequences using BLAST in the NCBI database. The sequence matched the sequence with accession number KAJ3901745.1, obtained by the conceptual translation of the *Lentinula edodes* genome (GenBank: MU818049.1). Taking into account this sequence and the amino-terminal sequence obtained by Edman degradation, specific primers were designed for the amplification of edodin cDNA. PCR fragments of approximately 1900 and 1400 bp were obtained (Figure S3) and cloned into the pCRTMII vector. Using other primer pairs, fragments were obtained and directly sequenced. The sequences of 13 overlapping fragments were obtained. Five of them showed the presence of introns, with consensus splicing sequences and stop codons within the intron, and eight of them lacked introns and presented an open reading frame. As previously reported, the retention of introns in mature mRNAs is a frequent phenomenon that could be related to some kind of regulation [25]. The sequenced cDNA consists of 1884 bp and contains 8 introns, which once removed produced a 1416 bp RNA encoding a protein of 472 amino acids (Figure 4), with a theoretical molecular mass of 52,147.52, which coincides with the molecular weight obtained from SDS-PAGE. Compared to the sequence KAJ3901745.1, edodin presents some differences: the first is the absence of 25 amino acids in the N-terminal region, which could be explained if the precursor is processed to an active protein through proteolytic cleavage. Also appearing is the replacement of leucine, arginine, and valine by proline, serine, and isoleucine, respectively, at positions 392, 393, and 397.

To confirm the amino acid sequence deduced from the edodin cDNA, and to verify the differences between the sequence KAJ3901745.1 and the one obtained in this work, a peptide-mapping strategy based on MALDI-ToF mass spectrometry (MS) was applied. After the purification of edodin and evaluation of its homogeneity by SDS-PAGE, the following experimental steps were taken for the peptide mapping of edodin: (i) hydrolysis of the protein by enzymatic (trypsin) and chemical (CNBr) cleavage; (ii) determination of the molecular masses of the peptides by MALDI-ToF MS analysis; and (iii) alignment of tryptic peptides with overlapping CNBr fragments. The exact molecular masses of the peptides obtained after tryptic digestion or cleavage with CNBr are reported in Table S1, while the overlapping peptides are shown in Figure S4 in comparison with the sequence deduced from the amplicons of this work. Overall, by using MALDI-TOF MS, a coverage of 76% of the edodin sequence was obtained (357 out of 472 amino acid residues) by considering the deduced cDNA sequence as reference. Although few overlapping peptides were found in the C-terminal region, probably due to their low solubility, this approach allowed us to verify the presence of proline, serine, and isoleucine residues at positions 392, 393, and 397, respectively, in the purified edodin.

### 2.5. Prediction of Edodin Structure

**Figure 5:**
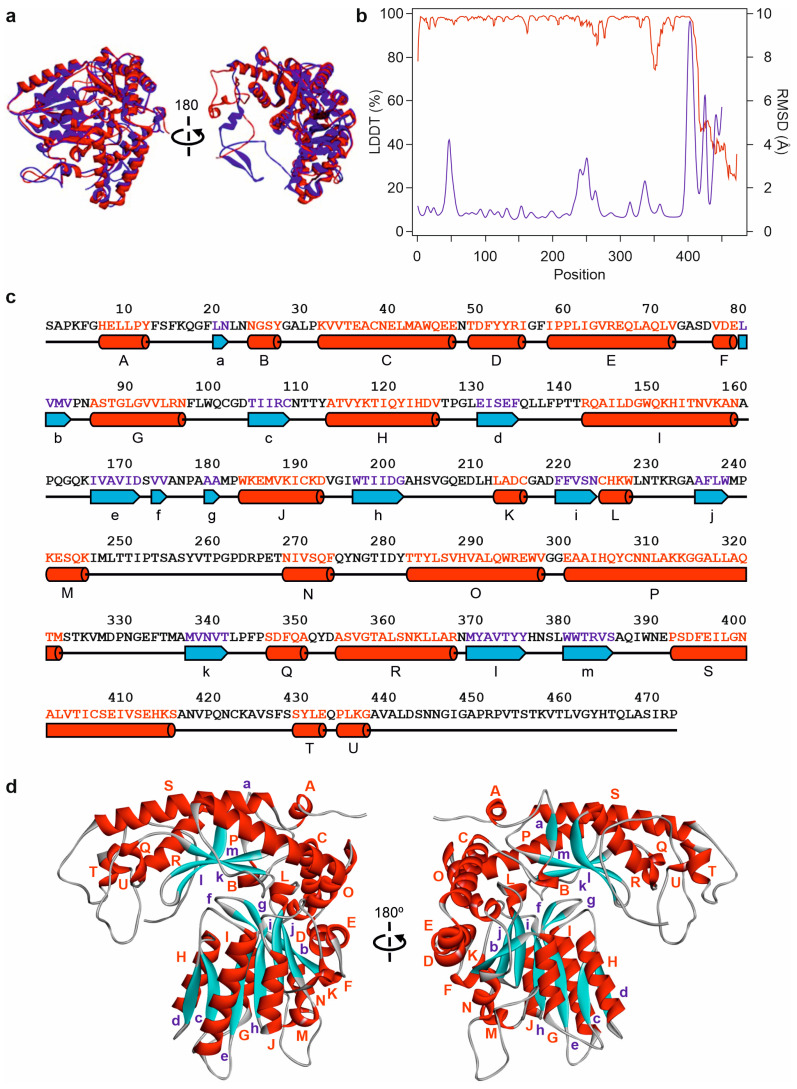
Structural predictions performed by AlphaFold2 (red) and RoseTTAFold (blue) (**a**); confidence tests (LDDT performed by AlphaFold2, red line; and RMSD performed by RoseTTAFold, blue line) are plotted (**b**); secondary (**c**) and three-dimensional (**d**) structures of edodin predicted by AlphaFold2 are depicted. α-Helices (red) are labeled A to U, and β-strands (cyan) are labeled a to m.

The programs AlphaFold2 [26] and RoseTTAFold [27] were used to predict the structure of edodin. As shown in Figure 5a, despite using different algorithms, the two programs predicted almost identical structures. The major differences between the two predictions occurred in the carboxyl-terminal region (residues 416–472), while the two predictions were consistent on the rest of the structure (Figure 5a). Both models showed excellent confidence indices, with LDDT values above 90% for the AlphaFold2 model and RMSD values below 1 Amstrong for most of the RoseTTAFold model (Figure 5b). This allows for these models to be used for studies at the molecular level [26]. The exception was the carboxyl-terminal region which showed values well below 50% for the AlphaFold2 model and well above 2 Amstrongs for the RoseTTAFold model (Figure 5b). These values suggest that this region could be an intrinsically disordered region (IDR) [28].

Edodin presents the characteristic structure of the fold type I of pyridoxal 5′-phosphate (PLP)-dependent enzymes [29,30]. Like other fold type I enzymes, edodin folds into a large domain (residues 24–298) consisting of a Greek-key αβα containing a seven-stranded mixed β-pleated sheet (b, j, i, h, e, c, and d) flanked by seven α-helices (E, K, J, I, H, G, and O) and several short β-strands and outside helices (Figure 5c,d). The small domain (residues 299–415) is an αβ fold with three antiparallel β-strands (k, l, and m) and three α-helices (P, R, and S). There is an N-terminal extension from the large domain (helix A) that is mainly associated with the small domain. There is also a C-terminal extension (residues 416–472) whose structure has not been solved by AlphaFold2 or RoseTTAFold, and which could be an intrinsically disordered region (IDR) [28].

The structural model shows predominantly α-helical secondary structures (212 out of 472 residues), which is in agreement with the far-ultraviolet circular dichroism spectrum of edodin, showing a protein profile with a predominance of α-helical elements considering two negative ellipticity signals at 222 and 208 nm and a positive ellipticity signal at 196 nm (Figure S5).

The structure predicted by AlphaFold2 has a high structural similarity to that of the kynureninase from *Pseudomonas fluorescens* (Figure S6), an enzyme with fold type I structure [30]. Although their sequences had only 21.45% identity, the TM-score value obtained with the TM-align algorithm [31] was 0.68165.

### 2.6. Putative Active Site of Edodin

**Figure 6:**
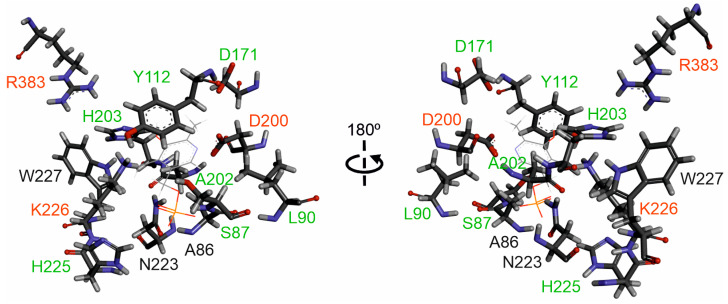
Prediction of the edodin active site. Amino acids potentially forming part of the edodin active site are represented by sticks, and the position occupied by PLP in *Pseudomonas fluorescens* kynureninase (Identifier 1QZ9) is represented by lines. The invariant amino acids in the fold type I of PLP-dependent enzymes are marked with red letters, amino acids specific to each enzyme family are marked with green letters, and amino acids that are not present in the other fold type I families are marked with black letters.

As discussed above, the confidence indices obtained in the edodin structure prediction allow, with appropriate caveats, studies at the molecular level. PLP-dependent enzymes exhibit diverse catalysis mechanisms and catalyze a wide variety of reactions, including oxidation–reduction, transfer of functional groups, hydrolysis, cleavage of various bonds, and isomerization [32]. Enzymes that present the fold type I have three invariant amino acids in the active site and between 10 and 12 residues that are specific to each enzyme family and are responsible for their functional diversity [33]. Edodin possesses the three invariable amino acids (D200, K226, and R383, Figure 6), which are important for catalytic activity and cofactor and substrate binding [33]. In addition, edodin has seven residues that are found in other enzyme families that exhibit the fold type I (S87, L90, Y112, D171, A202, H203, and H225, Figure 6). These residues participate in specific electrostatic and hydrogen bonding interactions with the PLP functional groups, which influences the cofactor properties to establish catalytic reactivity and reaction preference [33]. However, there are three amino acids (A86, N223, and W227) that are not present in the other families with the fold type I and that could be specific for edodin (Figure 6).

### 2.7. Homology of Edodin with Other Hypothetical Proteins

**Figure 7:**
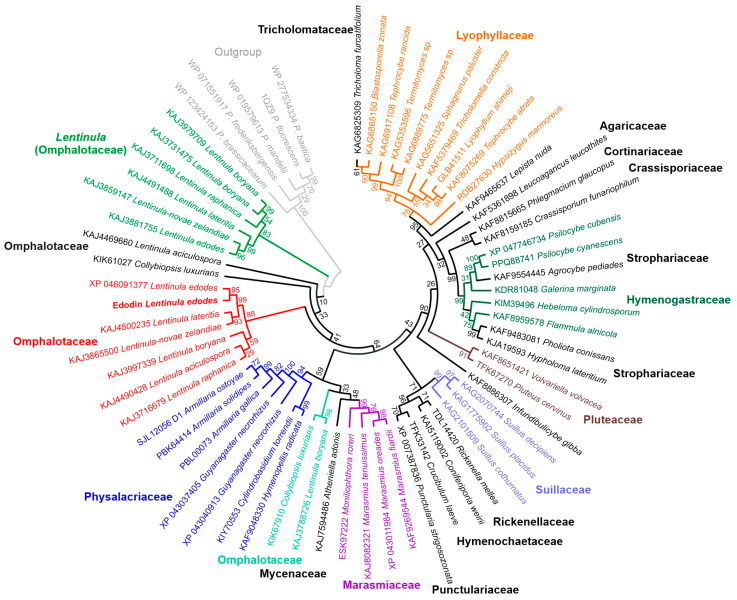
Phylogenetic analysis by maximum likelihood method of edodin and hypothetical proteins from several Agaricomycetes. Next to the branches, the percentage of trees in which the associated taxa are grouped is shown. The species and accession number are indicated, and next to them, in bold, is the family.

Using the edodin sequence, a database search yielded 100 hits, all of them sequences of unknown proteins (or described as pyridoxal phosphate-dependent transferases) obtained by a conceptual translation of the genomes or transcriptomes from fungi of the class Agaricomycetes (mainly from fungi of the order Agaricales, but also Hymenochaetales, Boletales, and Corticiales). Some were sequences from the same species, identical or with very few changes, and once the sequences from the same species with more than 95% identity were removed, 61 sequences were obtained and used for the phylogenetic study shown in Figure 7. The closest in the phylogenetic tree is sequence XP_046091377 from *Lentinula edodes* (Berk.) Pegler, which is almost identical to edodin, changes only one amino acid (P392L), and has 25 additional amino acids at the amino-terminal end. The latter difference is probably due to the processing of the precursor. Other sequences of species of the genus *Lentinula*, i.e., *Lentinula lateritia* (Berk.) Pegler, *Lentinula novae-zelandiae* (G. Stev.) Pegler, *Lentinula boryana* (Berk. and Mont.) Pegler, *Lentinula aciculospora* J.L. Mata and R.H. Petersen, and *Lentinula raphanica* (Murrill) Mata and R.H. Petersen (Figure 7), are placed in the same clade, whose sequences present percentages of identity with edodin between 84.53 and 98.52. In the phylogenetic tree, the paralogous sequence KAJ3881755 also appears, which has an identity percentage of 73.31% with edodin. In addition, other species of the genus *Lentinula* also show paralogs.

**Figure 8:**
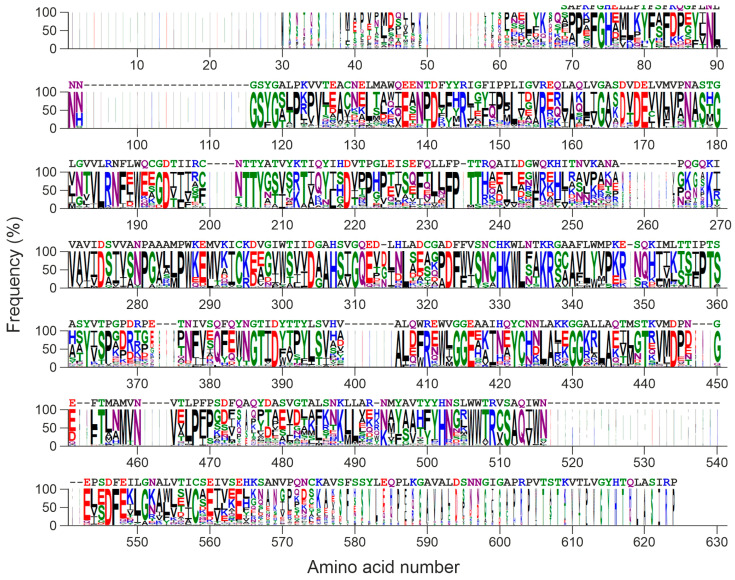
Sequence logo representation of the alignment of the sequences of Figure 7. The height of the letters is proportional to the frequency of the amino acid; and the width of the letter is proportional to their frequency but including gaps. The edodin sequence is represented above the logo. Colors indicate the chemical properties of the amino acids: polar (green), neutral (purple), hydrophobic (black), basic (blue), acidic (red).

Figure 8 shows a logo obtained with the alignment of the 61 sequences shown in Figure 7. Fifty-two amino acids remain unchanged, including almost all the amino acids mentioned in the previous section (S178, Y207, D275, D304, A306, H307, H330, K331, W332, and R509 in the logo) with the exceptions of L90 (181 in the logo) which can be V, L, I, or M, and A86 (177 in the logo) and N223 (328 in the logo), which are, however, the most frequent. This points to a conserved active site different from the other fold type I families.

## 3. Discussion

The study on cytotoxic proteins that are present in some fungi has always aroused great interest, on the one hand, to understand and avoid the risks that their consumption may entail, and on the other hand, because these proteins may have important biotechnological applications [34,35]. Some cytotoxic proteins are potent inhibitors of protein synthesis, such as ribosome-inactivating proteins (RIPs) [1,3,24], ribotoxins [11], and ribotoxin-like proteins (RL-Ps) [8]. This makes them promising tools for the construction of immunotoxins, conjugates, or engineered proteins for experimental therapy of cancer or viral diseases [15,16]. RIPs have also been used to obtain transgenic plants resistant to viruses, fungi, and insects [14,15]. RIPs are typical of plants and some bacteria [3,6], while ribotoxins are found only in ascomycete fungi [11] and RL-Ps only in basidiomycete fungi [8].

Three ribonucleases have been described in shitake fruiting bodies [36,37] and, recently, an RIP named ledodin [9]. Ledodin is a 22 kDa cytotoxic protein consisting of a single chain of 197 amino acids. It is a potent inhibitor of protein synthesis because it possesses N-glycosylase activity on the SRL of mammalian 28S rRNA. However, it is not active against insect, fungal, and bacterial ribosomes. Although it probably follows a catalytic mechanism similar to that of plant RIPs, no relation has been found between the sequence and structure of ledodin and any protein with known function, although many fungi have homologous sequences in their genome [9].

Ledodin was purified by a method used for the purification of plant RIPs [9]. An acidified crude extract was subjected to ion exchange chromatography on an SP-Sepharose column. After loading the sample, the column was washed with sodium acetate and sodium phosphate, and finally eluted with NaCl. However, the fraction resulting from washing with sodium phosphate (Figure 1a) also had protein synthesis inhibitory and rRNA N-glycosylase activities. This fraction, after being subjected to molecular exclusion chromatography (Figure 1b), yielded a 50 KDa protein which we named edodin.

Edodin inhibits protein synthesis in the rabbit reticulocyte lysate system but is 93-fold less active than ledodin (Figure 1e). An electrophoresis of the ribosomal RNA obtained from edodin-treated ribosomes allowed us to elucidate its enzymatic activity. Edodin is neither a ribonuclease nor a ribotoxin since no fragment(s) appear(s) on electrophoresis when ribosomes have been treated with edodin (Figure 2a). However, when the RNA is treated with acid aniline, which breaks the phosphodiester bond of the apurinic nucleotide [8,21], a fragment identical to that produced by BE27 and ledodin appears (Figure 2a), clearly indicating that edodin possesses N-glycosylase activity that acts on SRL.

Like ledodin, and unlike BE27, edodin was inactive on the ribosomes of mealworm, yeast, and *M. lysodeikticus* (Figure S1a–c). The GTPase-associated center (GAC) consists of the SRL, which is conserved in all living organisms, and the ribosomal stalk, which, in turn, consists of the conserved proteins of the base and the non-conserved proteins of the lateral elements [5,38]. These are the docking point of RIPs and, therefore, the sensitivity of the ribosomes depends on whether or not the toxins interact with the proteins of the lateral elements [5]. The fact that bacterial and yeast ribosomes are not sensitive to edodin has advantages and disadvantages. The disadvantage is that they cannot be used against diseases (either human or in crops) caused by fungi and bacteria, but it has the advantage that they can be cloned and expressed in the most commonly used microorganisms for this purpose.

Some RIPs from plants can also remove more than one adenine from 28S rRNA and also from other RNAs and DNA. Thus, RIP activity was also named adenine polynucleotide glycosylase (APG) [22,23]. RIPs from plants show different APG activities. Some of them have low activity, while others, such as BE27 or the RIPs obtained from *Phytolacca dioica*, have very high activity. Edodin, like ledodin [9], has little or no activity compared to BE27 (Figure S1). However, like BE27 [22] and unlike ledodin, it displays endonuclease (nicking) activity, promoting the conversion of supercoiled pCR2.1 DNA into the relaxed and linear forms (Figure 2b). This ability may be necessary for these proteins to perform different biological functions, including resistance to viruses [15].

Another difference in the activity of edodin and ledodin is their toxicity to different cell types. Ledodin is highly toxic to HeLa cells, but it is 300 times less toxic to COLO 320 cells [9]. Two things are striking about edodin: that despite being almost 100 times less active in the cell-free protein synthesis system, it is almost equally toxic to HeLa cells (IC_50_ is only 4 times higher, Figure 3a [9]), and that, unlike ledodin, it is also very toxic to COLO 320 cells (Figure 3a). The mechanism of cell toxicity is also slightly different, as ledodin mainly induces apoptosis [9], while for edodin, apoptosis seems to be a minority pathway (Figure 3b). This can be due to the fact that the cellular receptor binding and endocytosis pathway are different for the two proteins, since HeLa and COLO 320 cells do not identically express plasma membrane receptors. The sensitivity of different cell lines to toxins depends largely on the expression of receptors that RIPs use to internalize and reach within the cell the particular membrane where the translocation of the toxin to the cytosol occurs [39,40]. However, neither has an effect on the growth of *Penicilium digitatum*, (Figure S2) which may be attributed to the fact that the fungal ribosomes are not sensitive to either toxin (Figure S1b).

The size, amino acid sequence, and structure of edodin is very different from that of other plant and fungal rRNA N-glycosylases. Type 1 RIPs and the A-chain of type 2 RIPs from plants have a size of about 30 kDa, homologous sequences, and very similar structures, with invariant amino acids in the active site [41]. Fungal RIPs with rRNA N-glycosylase activity have different sizes (between 9.5 and 39 kDa), sequences, and probably different mechanisms [8]. Edodin is 50 kDa in size, and both AlphaFold2 and RoseTTAFold predict a structure related to the fold type 1 of PLP-dependent enzymes (Figure 4). PLP-dependent enzymes are the most prolific enzymes in metabolism as many of the reactions of metabolism are catalyzed by this superfamily of enzymes. The functional diversity of these enzymes is illustrated by the fact that more than 140 different enzyme activities listed by the Enzyme Commission are PLP-dependent, corresponding to approximately 4% of all classified activities [32]. Within fold type 1, there are isomerases (racemases), lyases (decarboxylases, aldolases), transferases (aminotransferases), and hydrolases (kyruneninase) that act on different types of bonds [33]. The similarity to the fold type 1 enzymes is confirmed by the presence of invariant amino acids in the active site, including K226, which forms the internal aldimine with PLP (Figure 6, [33]). Although the first step in the catalysis of this family is the formation of an external aldimine, the possible subsequent mechanisms are very diverse [33], so it would be very risky to venture how the action of edodin on SRL occurs on the ribosome.

The presence of homologous sequences in other fungi, some edible, (Figure 7) indicates that similar proteins are widespread in the fungal kingdom (Agaricomycetes) and the comparison of the sequences (Figure 8) points to a conserved structure. Recently, a search for sequences related to RIPs from plants and bacteria was carried out in the genomes of 1506 fungal species, and 46 sequences were found in 39 species, 38 ascomycetes, and 1 basidiomycete [42]. The only sequence found in basidiomycetes presents all the characteristic amino acids of the active site of RIPs from plants, although it is considerably smaller in size than RIPs from plants, bacteria, and ascomycetes [42]. Although their evolutionary origin, i.e., vertical or horizontal gene transfer, cannot be pinpointed [42], the presence of sequences related to RIPs from plants in the fungal genomes and of non-homologous rRNA N-glycosylases opens a new research subject to try to understand their function and biological role and to exploit their biotechnological potential.

On the other hand, similar sequences with L-cysteine sulphoxide lyase [43] and cysteine desulfurase [44] activities have been reported in *L. edodes*. However, these proteins differ from edodin in several aspects such as (i) molecular weight, (ii) isoelectric point, (iii) affinity for ion exchange columns, and (iv) cytotoxicity. However, the relationship between the activities of these proteins remains to be investigated.

## 4. Conclusions

Shiitake, one of the most consumed mushrooms worldwide, contains two cytotoxic proteins in the fruiting bodies. Ledodin and a new one present in large quantities, which we have named edodin. This protein is related to plant RIPs since it inhibits protein synthesis in a mammalian cell-free system and exhibits N-glycosylase activity on ribosomes by depurinating the sarcin–ricin loop (SRL) in the 28S rRNA. However, it is not capable of depurinating insect, fungal, and bacterial ribosomes. In addition, like many plant RIPs, it displays DNA nicking activity, although it does not possess significant adenine polynucleotide glycosylase activity. Surprisingly, in silico studies found no relationship between the sequence and structure of edodin and that of other RIPs. However, these studies predict that it has the characteristic structure of the fold type I of pyridoxal phosphate-dependent enzymes and, importantly, the enzyme edodin could be present in other fungi since, by searching the databases, proteins homologous to edodin have been found in the genome of several fungi, some of them edible, of the class Agaricomycetes.

The fact that a previously unknown family of cytotoxic proteins is present in some fungi is very important, both for food safety and for potential biomedical and biotechnological applications.

## 5. Materials and Methods

### 5.1. Reagents, Cells, and Fungi

The sources of the chemicals, cells, and fungi have been described previously [9]. Specific experimental details are given in the Supplementary Materials.

### 5.2. Methods

#### 5.2.1. Purification of Edodin

One hundred grams of fresh shiitake fruiting bodies were ground, extracted overnight, and acidified as described previously [9]. The acidified extract was subjected to cation exchange chromatography on an SP-Sepharose Fast Flow column equilibrated in 10 mM sodium acetate (pH 4.5) at a flow rate of 8.5 mL/min. After sample loading, the column was washed with 600 mL of 10 mM sodium acetate (pH 4.5) and eluted with 5 mM sodium phosphate (pH 6.66). The eluate (120 mL) was concentrated to 7 mL by ultrafiltration using an Amicon YM10 membrane and subjected to molecular exclusion chromatography on a HiLoad^®^ 26/600 Superdex^®^ 75 pg column (i.d. 2.6 × 60 cm, 320 mL) (GE Healthcare (Barcelona, Spain)) equilibrated with PBS at a flow rate of 2 mL/min. Fractions containing edodin (6.2 mg protein in 16 mL) were pooled, dialyzed in water, concentrated by ultrafiltration using an Amicon YM10 membrane to 4.6 mL, lyophilized into 0.5 mg aliquots, and stored at −20 °C until use. Ledodin was purified from the NaCl-eluted fraction on the SP-Sepharose column as described previously [9].

#### 5.2.2. Analytical Procedures

Determination of protein concentrations, sequencing of the amino-terminal end of edodin, SDS-PAGE of edodin, and glycosylation analysis were carried out as described previously [9,45]. Edodin circular dichroism analysis was performed as described elsewhere [46].

#### 5.2.3. Cell-Free Protein Synthesis and Enzyme Activity Assays

The effect of toxins on protein synthesis was determined by a coupled in vitro transcription–translation assay using a rabbit reticulocyte lysate system as described elsewhere [9]. The data represent the average of three experiments in duplicate.

Ribosomal RNA N-glycosylase assays in lysates of rabbit reticulocytes, mealworms, yeast, and bacteria as well as the measurement of adenine polynucleotide glycosylase (APG) activity in salmon sperm DNA and nicking activity experiments were performed according to the methods described elsewhere [9].

#### 5.2.4. Cell Viability and Antifungal Activity Assays

The viability of HeLa and COLO 320 cells was determined with a colorimetric assay based on the cleavage of WST-1 tetrazolium salt in formazan by mitochondrial dehydro-genases in viable cells, as described previously [9].

*P. digitatum* growth inhibition assays were performed in 96-well microtiter plates as described elsewhere [9].

#### 5.2.5. cDNA Synthesis, Cloning, and Sequencing

A portion of the shiitake fruiting body (100 mg) was ground in liquid nitrogen to a fine powder, and total RNA was isolated. Poly(A)-rich RNA was reverse transcribed using the synthetic oligonucleotide T1 (5′ CGTCTAGAGTCGAGTCGACTAGTGC(T)20 3′) following a procedure described previously [9]. Sequence-specific primers for the edodin gene were designed based on the N-terminal sequence, obtained by Edman degradation, and the sequence KAJ3901745.1: LE-F1 (5′ TCTGCCCCAAAATTCGGACACACG 3′) as forward primer, and LE-R1 (5′ AGGCCTAATGGAAGCAAGCAAGCTGC 3′) as reverse primer. cDNA amplification was carried out as described previously [9]. PCR amplification was performed under the following conditions: an initial denaturation at 94 °C 3 min, followed by 35 cycles of 94 °C 30 s, 54 °C 30 s, and 72 °C 90 s, and an additional 10 min extension at 72 °C. A PCR product of approximately 1900 bp (Figure S3a) was obtained, purified, and ligated into the pCRTMII vector and subsequently used to transform chemically competent E. coli INVαF’ bacteria. Five clones were purified and sequenced. The complete sequence of the gene coding for edodin with introns was obtained and, to rule out possible contamination of genomic DNA in the RNA preparation, a second RNA preparation was performed using the NucleoSpin^®^ RNA kit (Macherey-Nagel, Düren, Germany); this procedure includes a DNase treatment to completely remove any traces of genomic DNA. Reverse transcription and amplification were performed using T1, LE-F1, and LE-R1 primers under the same conditions as described above. A PCR product of approximately 1400 bp (Figure S3b) was obtained and purified. Additionally, several amplifications were performed using different primers located at internal positions of the sequence, and the amplicons obtained in each case were purified and sequenced. In total, 8 PCR products corresponding to overlapping sequences were sequenced to obtain the edodin coding cDNA without introns. The cDNA sequence for edonin was submitted to GenBank (accession number: PP003298).

#### 5.2.6. Edodin Cleavage and MALDI-ToF MS Analysis

Edodin cleavage and MALDI-ToF MS analysis were carried out as described previously [9,47].

#### 5.2.7. Prediction of Edodin Structure

Edodin structure was predicted using the structure prediction tools AlphaFold2 [26] and RoseTTAFold [27]. From the five models proposed by each program, those with the best prediction parameters were chosen. The alignment and comparison of protein structures was performed with the TM-align algorithm [31] on the website https://zhanggroup.org/TM-align/ (accessed 12 July 2023). Study representations and graphs of protein structures were constructed with the help of the Discovery Studio Visualizer suite (v21.1.0) (https://www.3dsbiovia.com/) (accessed 26 April 2022).

#### 5.2.8. Sequence Alignment and Phylogenetic Analysis

Amino acid sequences were obtained from the National Center for Biotechnology Information (https://www.ncbi.nlm.nih.gov/protein/) (accessed 20 February 2024). Sequence alignment and logo plotting were performed as indicated in [9] using MEGA 11 [48] and WebLogo 3 [49] software. Phylogenetic analysis was performed with MEGA 11 software [48] using the maximum likelihood method [50]. Fungi were named and classified according to the Index Fungorum (http://www.indexfungorum.org/) (accessed 20 February 2024).

## Data Availability

Data are available upon request; please contact the contributing authors.

## Supplementary Materials

The following supporting information can be downloaded at: https://www.mdpi.com/article/10.3390/toxins16040185/s1, Description of Materials and Methods; Figure S1: Ribosomal RNA N-glycosylase activity of edodin compared to BE27 in mealworm, yeast, and bacterial ribosomes and adenine polynucleotide glycosylase activity in salmon sperm; Figure S2: Antifungal activity of α-sarcin, BE27, and edodin against *Penicillium digitatum*; Figure S3: PCR amplification of the edodin cDNA; Table S1: Molecular mass values of trypsin peptides and chemical CNBr fragments of edodin determined by MALDI-ToF mass spectrometry after protein in-gel digestion; Figure S4: Overlapping peptides and chemical fragments used to map the amino acid sequence deduced from the cDNA sequence shown in Figure 4; Figure S5: Far-UV CD spectrum of edodin; Figure S6: Superposition of the structures of edodin and kynureninase from *Pseudomonas fluorescens* (Identifier 1QZ9).
