# Supplementary material for: Edodin: A New Type of Toxin from Shiitake Mushroom (Lentinula edodes) That Inactivates Mammalian Ribosomes
Source: Toxins (Basel). 2024 Apr 10;16(4):185. doi: 10.3390/toxins16040185 (PMC11053714; doi:10.3390/toxins16040185)
Supplement: Supplementary file 1 [file toxins-16-00185-s001.zip › toxins-2916686-supplementary.pdf]

The shiitake mushroom (*Lentinula edodes*) possesses a new type of toxin that inactivates mammalian ribosomes

Lucía Citores, Sara Ragucci, Claudia C. Gay, Rosita Russo, Angela Chambery, Antimo Di Maro, Rosario Iglesias and José M. Ferreras

MATERIALS AND METHODS

Materials

Shiitake (*Lentinula edodes* (Berk.) Pegler) fruiting bodies, from the company Hongos Fernández Guridi (Pradejón, La Rioja, Spain) and mealworms (Tenebrio molitor L.) were purchased from local markets. The *Penicillium digitatum* (Pers.) Sacc. strain was obtained from the Spanish Type Culture Collection (CECT), Valencia, Spain. The sources of the chemicals were described previously [22]. SP-Sepharose was purchased from GE Healthcare (Barcelona, Spain). TPCK-treated trypsin (sequencing grade) was purchased from Merck Life Science S.r.l. (Milan, Italy). HPLC grade solvents were obtained from Merck (VWR International S.r.l., Milan, Italy). Cyanogen bromide (CNBr) was obtained from Fluka (Milan, Italy). Century™-Plus RNA markers were purchased from Fisher Scientific (Madrid, Spain). Potato dextrose agar (PDA) and potato dextrose broth (PDB) media were purchased from Sigma-Aldrich (Madrid, Spain).

Cell lines and culture

COLO 320 (human colorectal adenocarcinoma) and HeLa (human epitheloid cervix carcinoma) cells were obtained from the European Culture Collection (ECACC) and cultured in RPMI 1640 medium (GIBCO BRL, Barcelona, Spain) supplemented with 10% foetal bovine serum (FBS), 100 U/mL penicillin and 0.1 mg/mL streptomycin under 5% CO2 at 37 °C.

Purification of edodin and ledodin

One hundred grams of fresh shiitake fruiting bodies were ground with a blender and extracted overnight at 4 °C with eight volumes of PBS (140 mM NaCl, containing 5 mM sodium phosphate, pH 7.5). The extract was clarified by filtering through a nylon mesh and then centrifuging for 30 minutes at 9,000 rpm in a rotor Beckman JA-10 (12,900 g) at 2 °C. Glacial acetic acid was added to the supernatant until a pH of 4,0 was reached, and it was clarified again by filtration and centrifugation under the same conditions. The acidified extract was subjected to cation exchange chromatography on a SP-Sepharose Fast Flow column (i.d. 5 × 8 cm, 157 mL) equilibrated in 10 mM sodium acetate (pH 4,5) at a flow rate of 8,5 mL/min. After sample loading, the column was washed with 600 mL of 10 mM sodium acetate (pH 4.5) and eluted with 5 mM sodium phosphate (pH 6.66). The eluate (120 mL) was concentrated to 7 mL by ultrafiltration using an Amicon YM10 membrane and subjected to molecular exclusion chromatography on a HiLoad® 26/600 Superdex® 75 pg column (i.d. 2.6 × 60 cm, 320 mL) equilibrated with PBS at a flow rate of 2 mL/min. Fractions containing edodin (6.2 mg protein in 16 mL) were pooled, dialysed in water (3 times in 4 L water at 5 °C), concentrated by ultrafiltration using an Amicon YM10 membrane to 4.6 mL, lyophilised into 0.5 mg aliquots and stored at -20 °C until use. Ledodin was purified from the NaCl-eluted fraction on the SP-Sepharose column as described previously [9].

Analytical procedures

Protein concentrations were determined using a spectrophotometric method [45]. Homogeneity of the isolated protein was determined by SDS-PAGE with a Mini-Protean II device (Bio-Rad; Milan, Italy), using 6% (w/v) stacking and 12% (w/v) separation gels under reducing conditions; a Precision Plus Protein™ kit (Bio-Rad) was used as the reference proteins. Glycosylation analysis was performed on the gel after SDS-PAGE using Pro-Q™ Emerald 300 Glycoprot Probes Kombo (Life Technologies Italia Fil., Monza, Italy). Glycosylated proteins were visualised using the ChemiDoc™ XRS system (Bio-Rad). The amino-terminal

end of edodin was sequenced by automated Edman degradation performed on a Procise 494 sequencer (Applied Biosystems Inc., Foster City, CA, USA) at the Service of Protein Chemistry at the Margarita Salas Center for Biological Research (CIBMS, Madrid, Spain).

Cell-free protein synthesis and enzyme activity assays

The effect of toxins on protein synthesis was determined by a coupled in vitro transcription-translation assay using a rabbit reticulocyte lysate system ~~as described elsewhere [4]~~. The reaction mixture contained 0.6 µL of rabbit reticulocyte lysate and 5.8 µL of a mixture of the following components: 4.6 U ribonuclease inhibitor, 2.3 U T7 RNA polymerase, 0.2 µg luciferase T7 plasmid, rNTPs (0.4 mM each), amino acids (2 µM each), 10 mM Tris–HCl (pH 7.8), 0.2 mM spermidine, 28 mM KCl, 1 mM MgCl2, and nuclease-free water. The mixtures were incubated at 30 ◦C for 10 min and placed on ice. Then, 1.6 µL of either water or different protein concentrations were added and the sample mixture was incubated at 30 ◦C for 40 min. Subsequently, 25 µL water was added and mixed with 28 µL of Luciferase Assay Reagent (Promega, Alcobendas, Madrid, Spain) at room temperature. Luminescence was determined with a Junior LB 9509 luminometer (Berthold Technologies GmbH & Co. KG, Bad Wildbad, Germany). The mean results ± SE of three experiments performed in duplicate are reported. IC50 (concentration that inhibits 50% protein synthesis) values were calculated by linear regression.

Ribosomal RNA N-glycosylase assays on lysates of rabbit reticulocytes, mealworms, yeast and bacteria were performed as described elsewhere [22,9].

Adenine polynucleotide glycosylase (APG) activity on salmon sperm DNA was measured according to the method reported elsewhere [22] with some modifications. Ten µg of salmon sperm DNA was incubated with 10 µg of toxin in 300 µL of a reaction mixture containing 100 mM KCl and 50 mM magnesium acetate (pH 4), at 30 °C for 2 h. After incubation, the DNA was precipitated with ethanol at -80 °C overnight and centrifuged at 13,000 rpm for 15 min. The amount of nucleobases released from the toxin-treated DNA was determined by measuring the absorbance of the supernatant at 260 nm.

Nicking activity experiments were performed as previously reported [5]. Each reaction contained 5 µg of toxin and 100 ng of pCR2.1 DNA in a final volume of 10 µL of 10 mM Tris-HCl (pH 7.8), 50 mM NaCl and 50 mM KCl. Samples were incubated for 2 h at 30 °C in the absence or presence of 5 mM MgCl2, run on a 0.8 % (w/v) agarose gel in TAE buffer (0.04 M Tris, 0.04 M acetate, 1 mM EDTA, pH 8.0) and visualised by nucleic acid staining with Gel Red (Biotium Inc. , Hayward, CA).

Cell viability and antifungal activity assays

The viability of HeLa and COLO 320 cells was determined with a colorimetric assay based on the cleavage of WST-1 tetrazolium salt in formazan by mitochondrial dehydrogenases in viable cells, as described elsewhere [9].

Growth inhibitory assays of toxins against *P. digitatum* were performed in 96-well microtitre plates. Conidia of *P. digitatum* (100 spores/well) were incubated at 26 °C in 150 µL of PDB medium in the presence of different concentrations of toxins. Fungal growth was monitored spectrophotometrically using a microtitre plate Multiskan EX reader (Thermo Scientific, Waltham, MA, USA) after different times of incubation. Each experiment was performed in triplicate.

cDNA synthesis, cloning and sequencing

A portion of the shiitake fruiting body (100 mg) was broken with a mortar and pestle and ground in liquid nitrogen to a fine powder, and total RNA was isolated with the RNeasy Minikit (Qiagen, Barcelona, Spain) according to the manufacturer's instructions. Poly(A)-rich RNA was reverse transcribed using the synthetic oligonucleotide T1 (5' CGTCTAGAGTCGAGTCGACTAGTGC(T)20 3'). One µg of total RNA was incubated with 1 µL of RNase inhibitor at 65 °C in a thermal cycler for 5 min. It was then cooled on ice for 1 min and 15 µL of a reaction mixture (1× PCR Buffer II, 5 mM MgCl2, 1 mM of each dNTP, 10 µM of T1, and 2.5 units of MuLV reverse transcriptase; RNA PCR kit Roche, Roche Diagnostics SL, Sant Cugat del Vallès, Barcelona, Spain) was added. The reaction mixture was incubated for 20 min at 23 °C, then 20 min at 42 °C and finally

5 min at 99 °C. Sequence-specific primers for the edodin gene were designed and synthesized based on the N-terminal sequence, obtained by Edman degradation, and the sequence KAJ3901745.1: LE-F1 (5' TCTGCCCCAAAATTCGGACACACG 3') as forward primer, and LE-R1 (5' AG-GCCTAATGGAAGCAAGCAAGCTGC 3') as reverse primer. For cDNA amplification, 0.2 μL of the previously synthesised cDNA and 18.8 μL of the master mix (1× PCR buffer/Mg2+, 0.2 mM dNTPs mix, 2.5 units of Taq Polymerase (Invitrogen, Madrid, Spain) and 0.5 μM of each primer) were used. PCR amplification was performed under the following conditions: an initial denaturation at 94 °C 3 min, followed by 35 cycles of 94 °C 30 s, 54 °C 30 s and 72 °C 90 s, and an additional 10 min extension at 72 °C. Approximately 5 μL of the amplified products were analysed on 0.8% (w/v) agarose gel. A PCR product of approximately 1900 bp (Figure S3a) was obtained and purified using the NucleoSpin® Gel and PCR Clean-up kit (Macherey-Nagel, Düren, Germany), according to the manufacturer's instructions. The purified PCR fragment was ligated into the pCRTMII vector and subsequently used to transform chemically competent E. coli INVαF′ bacteria (Invitrogen-Thermo Fisher Scientific, Alcobendas, Spain) according to the manufacturer's instructions. Five clones were purified and sequenced using M13 primers. The complete sequence of the gene coding for edodin with introns was obtained and, to rule out possible contamination of genomic DNA in the RNA preparation, a second RNA preparation was performed using the NucleoSpin® RNA kit (Macherey-Nagel, Düren, Germany); this procedure includes a DNase treatment to completely remove any traces of genomic DNA. Reverse transcription and amplification were performed using T1, LE-F1 and LE-R1 primers under the same conditions as described above. A PCR product of approximately 1400 bp (Figure S3b) was obtained and purified using the NucleoSpin® Gel and PCR Clean-up kit and sequenced. Additionally, several amplifications were performed using different primers located at internal positions of the sequence and the amplicons obtained in each case were purified and sequenced. In total, 8 PCR products corresponding to overlapping sequences were sequenced to obtain the complete sequence of the edodin coding cDNA without introns. DNA sequencing was carried out at Cenit Support Systems (Villamayor, Salamanca, Spain). The DNA sequence for edonin was submitted to GenBank (accession number: PP003298).

Edodin cleavage and MALDI-ToF MS analysis

For in situ trypsin digestion, Coomassie-stained protein bands (~20 μg) of edodin were excised from the gels and destained by washing twice with 400 μL of water and then with 50% acetonitrile. Gel pieces were dried in a SpeedVac Vacuum (Savant Instruments Inc., Saroor Nagar, Hyderabad, India) and rehydrated with 150 μL of 50 mM NH4HCO3, pH 8.0. Subsequently, the hydrated bands were subjected to disulphide reduction with DTT 10 mM (15 min at 55 °C) and alkylation with iodoacetamide 50 mM (15 min at room temperature in the dark). Enzymatic proteolysis was performed by incubating bands at 37 °C for 3 h following the addition of TPCK-treated bovine trypsin (140 ng). Peptides were extracted in two steps by sequential addition of 150 μL of 1% trifluoroacetic acid (TFA) and 50 μL of 2% TFA/50% acetonitrile for 10 min in a sonication bath. The combined supernatants were dried in a SpeedVac Vacuum and resuspended in 5.0 μL of 0.1% TFA/50% acetonitrile for further analysis. Chemical fragmentation of edodin (~100 μg) with cyanogen bromide (CNBr) was performed in 70% TFA to minimize adduct formation in the presence of formic acid, as described [47].

Mixture aliquots (1.0 μL) of either in situ tryptic digestion (T-) or chemical (CNBr) fragmentation (CB-) were mixed (1:1; v:v) with saturated α-cyano-4-hydroxycinnamic acid matrix solution (10 mg/mL in acetonitrile/water (1:1, v/v), containing 0.1% TFA) and spotted onto a matrix-assisted laser desorption ionization-time of flight (MALDI-ToF) micro MX (Waters, Milford, MA USA) target plate. Samples were air dried and then loaded into the mass spectrometer. Peptide spectra were collected in positive ion reflectron mode with the settings: source voltage, 12 kV; pulse voltage, 1999 V; detector voltage, 5200 V; and reflectron voltage, 2350 V. Measurements were performed in the mass range m/z 500-5000 with a suppression mass gate set to m/z 500. The instrument was calibrated externally using a tryptic alcohol dehydrogenase digest (Waters, Milford, MA, USA) as standard. Intact protein spectra were acquired in positive ion linear mode, by using a pulse voltage of 1200 V. A four-point external calibration was applied by using an appropriate mixture (10 pmol/μL) of insulin, cytochrome c, horse Mb and trypsinogen as standard proteins (Merk Life Science S.r.l.).

Edodin circular dichroism analysis

CD spectra were obtained at room temperature on a Jasco J-815 dichrograph (Jasco Europe, Cremella (LC) Italy). For the far-UV spectrum, measurements were performed with a protein concentration of 0.16 mg/mL in 10 mM Na-phosphate, pH 7.2, using a 0.1 cm path length quartz cuvette. DichroWeb (http://dichroweb.cryst.bbk.ac.uk/html-home.shtml) was used to estimate the percentage of secondary structural elements [46].

Prediction of edodin structure

Edodin structure prediction was performed with AlphaFold2 software [26] following the instructions on the website https://colab.research.google.com/github/sokrypton/ColabFold/blob/main/AlphaFold2.ipynb#scrollTo=G4yBrceuFbf3 (accessed 2 July 2023), and with RoseTTAFold software [27] following the instructions on the website https://robetta.bakerlab.org (accessed 6 July 2023). From the five models proposed by each program, those with the best prediction parameters were chosen. Alignment and comparison of protein structures was performed with the TM-align algorithm [31] on the website https://zhanggroup.org/TM-align/ (accessed 12 July 2023). Study representations and graphs of protein structures were constructed with the help of the Discovery Studio Visualizer suite (v21.1.0) (https://www.3dsbiovia.com/) (accessed 26 April 2022).

Sequence alignment and phylogenetic analysis

All amino acid sequences used in this study are available in the National Center for Biotechnology Information (NCBI) sequence database (https://www.ncbi.nlm.nih.gov/protein/). Sequence alignment was performed with the ClustalW tool included in the Mega 11 package (version 11.0.13; http://www.megasoftware.net (accessed 19 July 2023)) [48]. Multiple sequence alignment was graphically represented using a sequence logo created with WebLogo 3 (http://weblogo.threeplusone.com/ (accessed 30 June 2023)). Evolutionary analysis was inferred using the Maximum Likelihood method as described elsewhere [50] and performed in MEGA 11 [12]. Representative sequences of bacterial kynureninases were used as out-group. The name and classification of fungi were obtained from Index Fungorum (http://www.indexfungorum.org/).

References

9. Citores, L.; Ragucci, S.; Russo, R.; Gay, C.; Chambery, A.; Di Maro, A.; Iglesias, R.; Ferreras, J. Structural and functional characterization of the cytotoxic protein ledodin, an atypical ribosome-inactivating protein from shiitake mushroom (Lentinula edodes). Protein Science 2023, 32, doi:10.1002/pro.4621. 9

22. Iglesias, R.; Citores, L.; Ragucci, S.; Russo, R.; Di Maro, A.; Ferreras, J.M. Biological and antipathogenic activities of ribosome-inactivating proteins from Phytolacca dioica L. Biochimica Et Biophysica Acta-General Subjects 2016, 1860, 1256-1264, doi:10.1016/j.bbagen.2016.03.011.

26. Jumper, J.; Evans, R.; Pritzel, A.; Green, T.; Figurnov, M.; Ronneberger, O.; Tunyasuvunakool, K.; Bates, R.; Zidek, A.; Potapenko, A.; et al. Highly accurate protein structure prediction with AlphaFold. Nature 2021, 596, 583-589, doi:10.1038/s41586-021-03819-2.

27. Baek, M.; DiMaio, F.; Anishchenko, I.; Dauparas, J.; Ovchinnikov, S.; Lee, G.; Wang, J.; Cong, Q.; Kinch, L.; Schaeffer, R.; et al. Accurate prediction of protein structures and interactions using a three-track neural network. Science 2021, 373, 871-876, doi:10.1126/science.abj8754.

31. Zhang, Y.; Skolnick, J. TM-align: a protein structure alignment algorithm based on the TM-score. Nucleic Acids Research 2005, 33, 2302-2309, doi:10.1093/nar/gki524.

45. Kalb, V.F.J.; Bernlohr, R.W. A new spectrophotometric assay for protein in cell extracts. Analytical Biochemistry 1977, 82, 10, doi:10.1016/0003-2697(77)90173-7.

46. Whitmore, L.; Wallace, B. Protein secondary structure analyses from circular dichroism spectroscopy: Methods and reference databases. Biopolymers 2008, 89, 392-400, doi:10.1002/bip.20853.

47 Rodriguez, J.; Wong, L.; Jennings, P. The solvent in CNBr cleavage reactions determines the fragmentation efficiency of ketosteroid isomerase fusion proteins used in the production of recombinant peptides. Protein Expression and Purification 2003, 28, 224-231, doi:10.1016/S1046-5928(02)00700-3.

48. Tamura, K.; Stecher, G.; Kumar, S. MEGA11 Molecular Evolutionary Genetics Analysis Version 11. Molecular Biology and Evolution 2021, 38, 3022-3027, doi:10.1093/molbev/msab120.

50. Tamura, K.; Peterson, D.; Peterson, N.; Stecher, G.; Nei, M.; Kumar, S. MEGA5: Molecular Evolutionary Genetics Analysis Using Maximum Likelihood, Evolutionary Distance, and Maximum Parsimony Methods. Molecular Biology and Evolution 2011, 28, 2731-2739, doi:10.1093/molbev/msr121

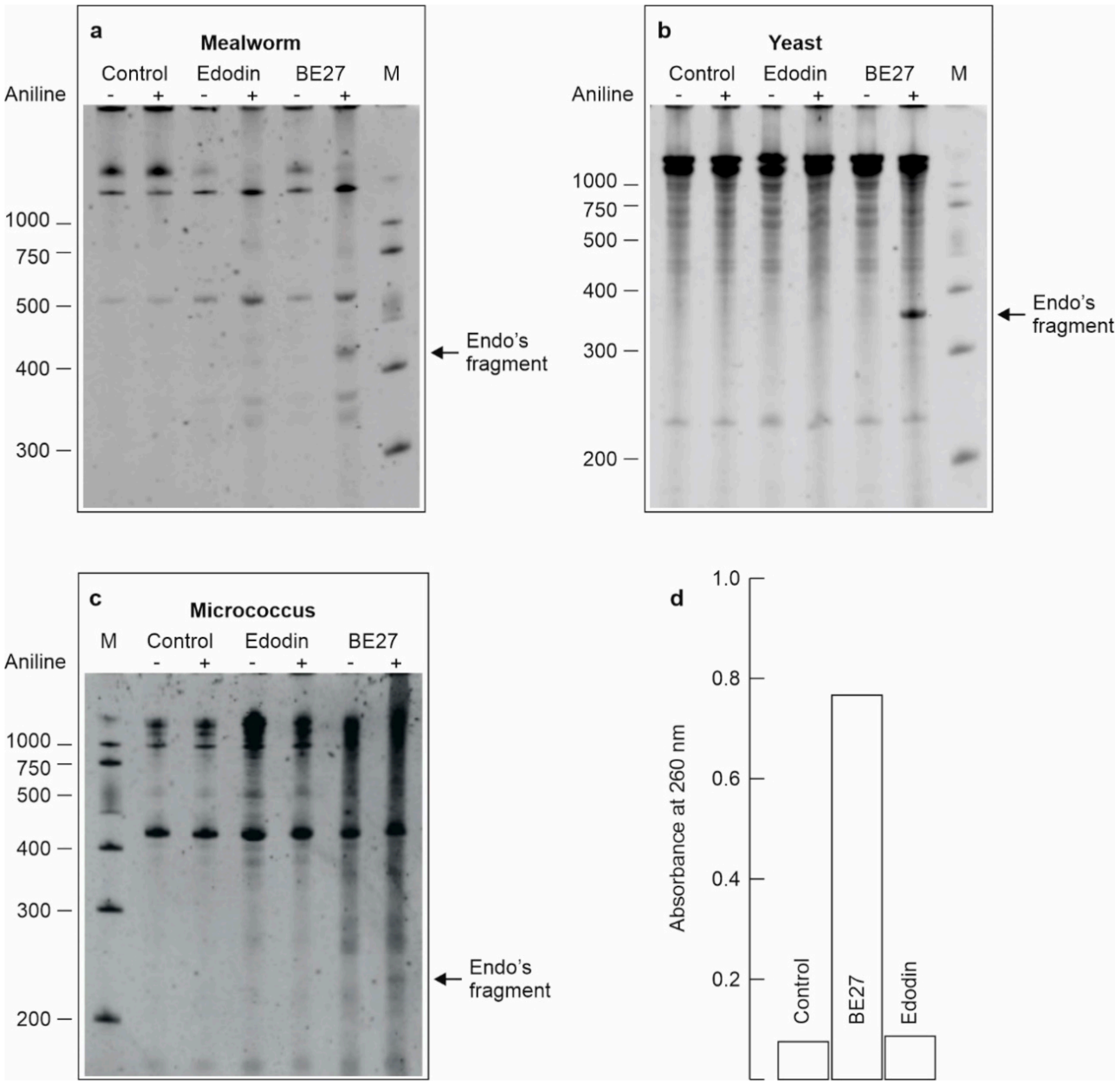

Figure S1. Ribosomal RNA N-glycosylase activity of edodin compared to BE27 in mealworm (**a**), yeast (**b**) and bacterial ribosomes (**c**) and adenine polynucleotide glycosylase activity in salmon sperm (**d**): The rRNA N-glycosylase activity was tested as indicated in Materials and Methods. Each lane contained RNA isolated from mealworm ribosomes (3 µg per lane, **a**) from the yeast *Saccharomyces cerevisiae* (5 µg per lane, **b**), and the bacterium *Micrococcus lysodeikticus* (1.5 µg per lane, **c**) untreated (control) or toxin-treated. Arrows indicate the RNA fragment (Endo's fragment) released as a result of toxin action after treatment with acid aniline (+). Numbers indicate the size of the markers in nucleotides; (**d**) The adenine polynucleotide glycosylase (APG) activity of 3 µg of edodin or BE27 was assayed on salmon sperm DNA as described in Materials and Methods and the absorbance of released nucleobases was measured at 260 nm. Data represent the mean of two duplicate experiments.

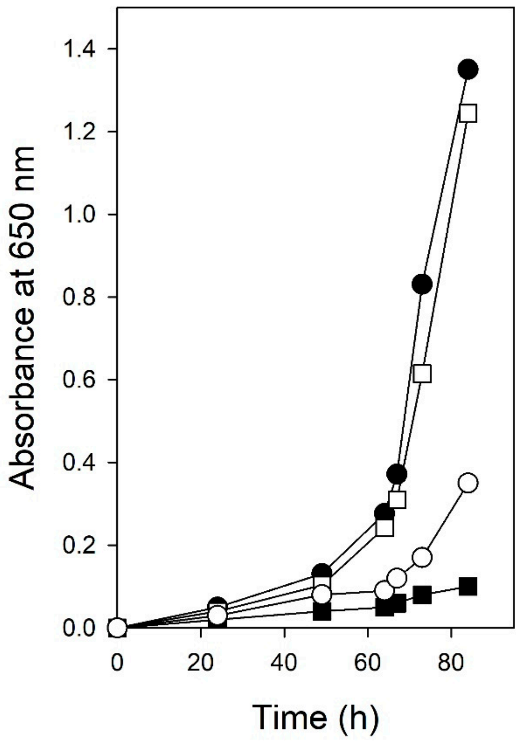

Figure S2 Antifungal activity of α-sarcin, BE27 and edodin against *Penicillium digitatum*. Conidia of *P. digitatum* were grown at 28 °C in PDB medium in the absence (filled circles) or in the presence of 1 µg/mL α-sarcin (filled squares), 15 µg/mL BE27 (open circles) and 100 µg/mL edodin (open squares). Fungal growth was measured as an increase in absorbance at 650 nm.

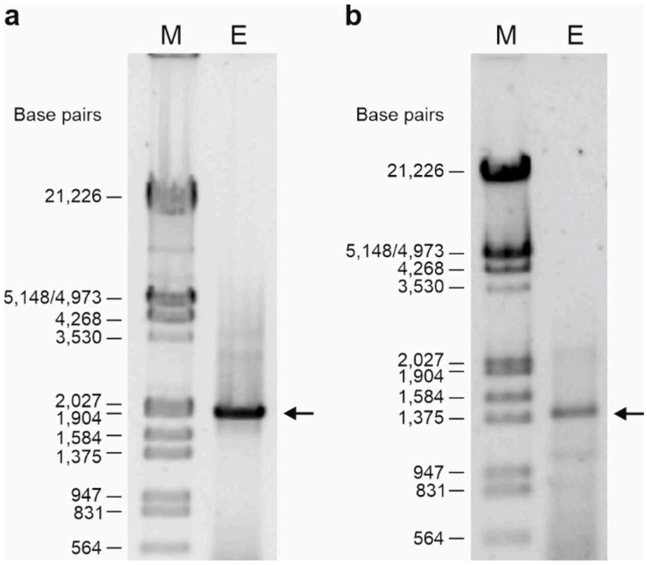

Figure S3 PCR amplification of the edodin cDNA (E). Amplicons with (**a**) and without (**b**) introns are shown. The arrow indicates the amplification product, and the numbers, the size of the markers (M, Lambda DNA-EcoR I/Hind III double digest) in base pairs.

Table S1. Molecular mass values of trypsin (T) peptides and chemical CNBr (CB) fragments of edodin determined by MALDI-ToF mass spectrometry after protein in-gel digestion.

Peptide	Sequence position	Experimental molecular mass[a]	Theoretical molecular mass[b]	*Δ* (Da)	Missed cleavage at	Notes
Trypsin peptides						
T-1	1-16	1867.87	1867.97	0.10	K4	*N-terminal*
T-1′	5-16	1484.70	1484.75	0.05		
T-2	17-32	1692.92	1692.87	0.05		
T-3	33-54	2768.45	2768.20	0.25		Cys CAM:38[c]
T-4	55-65	1181.73	1181.74	0.01		
T-5	96-117	2724.78	2724.29	0.49	R107	Cys CAM:101, 108[c]
T-5′	96-107	1522.74	1522.75	0.01		Cys CAM:101[c]
T-6	118-142	2918.83	2918.53	0.30		G124D
T-7	143-151	1054.53	1058.56	0.03		
T-7′	143-157	1750.93	1750.95	0.02	K151	
T-7′′	143-165	2545.48	2545.36	0.12	K151 and K157	
T-8	152-165	1505.85	1505.83	0.02	K157	
T-9	233-245	1491.52	1492.76	1.24	K241	
T-10	295-312	2060.96	2059.96	1.00		Cys CAM:307[c]
CNBr fragments						
CB-1[#]	1-42	4589.27	4586.22	3.05		
CB-2[#]	43-82	4539.71	4537.08	2.63		
CB-3	83-181	1792.58	1796.38	3.8		MSO[d]
CB-4[#]	182-239	6543.96	6543.54	0.42	M186	
CB-4′[#]	187-239	5886.01	5887.73	1.72		MSO[d]
CB-5[#]	248-327	8774.29	8774.86	0.57	M322	MSO[d]
CB-5′[#]	248-322	8226.32	8228.18	1.86		MSO[d]
CB-6[#]	338-369	3423.07	3421.83	1.24		

[a] $[M+H^+]^+$, experimental molecular mass values obtained by MALDI-TOF MS.

[c] Cysteine carbamidomethylation following reduction and S-Carboxymethylation with iodoacetamide, used to block cysteinyl residues from oxidation, preventing them from reacting and forming disulfide linkages. The position of S-Carboxymethylated cysteinyl residue is also indicated.

[d] MSO, methionine sulfoxide form of modified methionine.

[#] average molecular mass is reported, while for each of other peptides, the monoisotopic molecular masses have been considered.

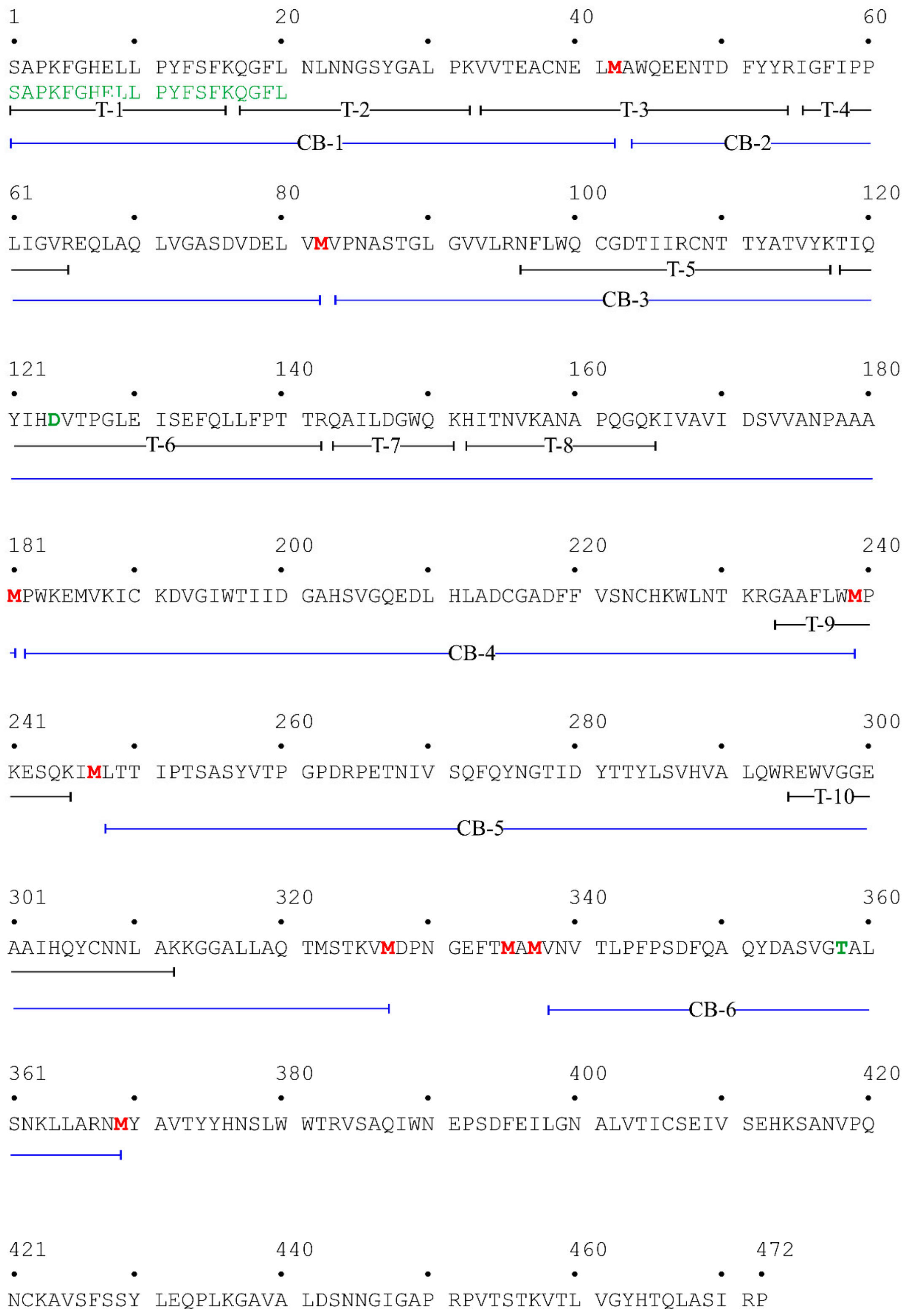

Figure S4 Overlapping peptides and chemical fragments used to map the amino acid sequence deduced from the cDNA sequence shown in Figure 4 (see Table S1 for details of peptide assignment). CB, CNBr fragments; T, tryptic peptides. The N-terminal sequence of the native protein obtained by Edman degradation is shown in green.

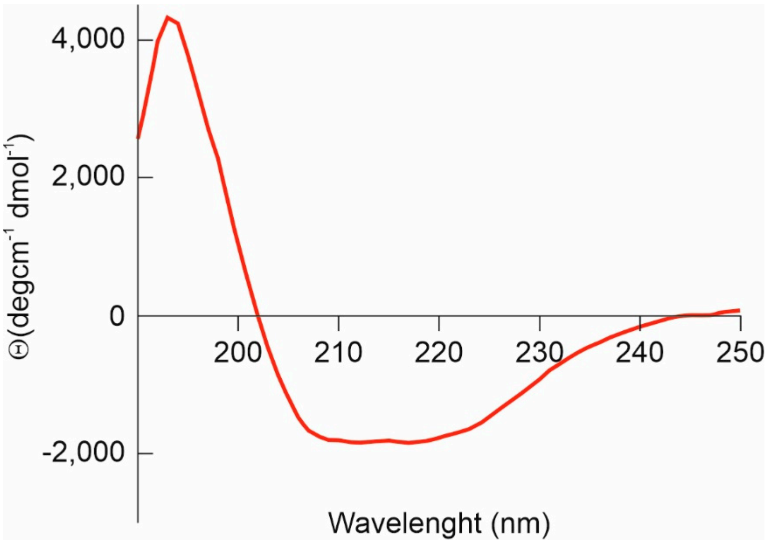

Figure S5 Far-UV CD spectrum of edodin.

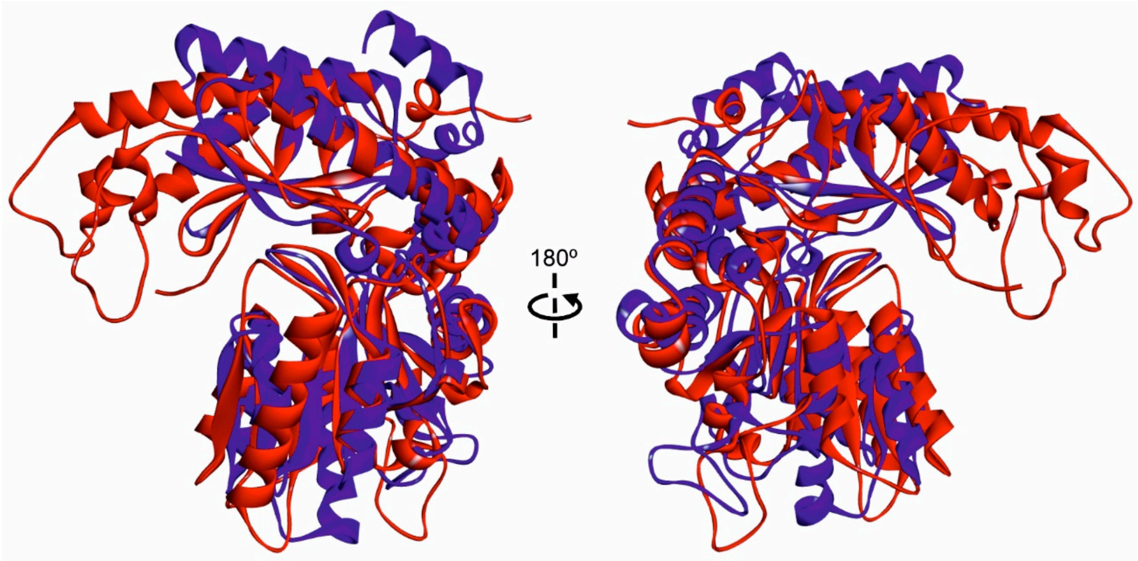

Figure S6 Superposition of the structures of edodin and kynureninase from *Pseudomonas fluorescens* (Identifier 1QZ9). The superposition was carried out with the TM-align algorithm, as described in Materials and Methods.
